# Effects of ACLY Inhibition on Body Weight Distribution: A Drug Target Mendelian Randomization Study

**DOI:** 10.3390/genes15081059

**Published:** 2024-08-12

**Authors:** Dipender Gill, Marie-Joe Dib, Rubinder Gill, Stefan R. Bornstein, Stephen Burgess, Andreas L. Birkenfeld

**Affiliations:** 1 Department of Epidemiology and Biostatistics, School of Public Health, Imperial College London, London W2 1PG, UK; 2 Primula Group Ltd., London N8 0RL, UK; rubinder@primulagroup.co.uk; 3 Division of Cardiovascular Medicine, Perelman School of Advanced Medicine, University of Pennsylvania, Philadelphia, PA 19104, USA; marie-joe.dib@pennmedicine.upenn.edu; 4 Department of Internal Medicine III, University Clinic, Technical University Dresden, D-01062 Dresden, Germany; stefan.bornstein@ukdd.de; 5 German Center for Diabetes Research (DZD), D-85764 Neuherberg, Germany; andreas.birkenfeld@med.uni-tuebingen.de; 6 Department of Diabetes, School of Cardiovascular and Metabolic Medicine & Sciences, King’s College London, London WC2R 2LS, UK; 7 MRC Biostatistics Unit, University of Cambridge, Cambridge CB2 0SR, UK; sb452@medschl.cam.ac.uk; 8 Department of Public Health and Primary Care, University of Cambridge, Cambridge CB2 0SR, UK; 9 Department of Internal Medicine IV, Diabetology, Endocrinology and Nephrology, Eberhard Karls University Tübingen, D-72074 Tübingen, Germany; 10 Institute for Diabetes Research and Metabolic Diseases, Helmholtz Center Munich, Eberhard Karls University Tübingen, D-72074Tübingen, Germany

**Keywords:** ACLY, metabolic disease, type 2 diabetes mellitus, Mendelian randomization

## Abstract

**Background:** Adenosine triphosphate-citrate lyase (ACLY) inhibition has proven clinically efficacious for low-density lipoprotein cholesterol (LDL-c) lowering and cardiovascular disease (CVD) risk reduction. Clinical and genetic evidence suggests that some LDL-c lowering strategies, such as 3-hydroxy-3-methylglutaryl-coenzyme A reductase (HMGCR) inhibition with statin therapy increase body weight and the risk of developing type 2 diabetes mellitus (T2DM). However, whether ACLY inhibition affects metabolic risk factors is currently unknown. We aimed to investigate the effects of ACLY inhibition on glycaemic and anthropometric traits using Mendelian randomization (MR). **Methods:** As genetic instruments for ACLY inhibition, we selected weakly correlated single-nucleotide polymorphisms at the *ACLY* gene associated with lower *ACLY* gene expression in the eQTLGen study (N = 31,684) and lower LDL-c levels in the Global Lipid Genetic Consortium study (N = 1.65 million). Two-sample Mendelian randomization was employed to investigate the effects of ACLY inhibition on T2DM risk, and glycaemic and anthropometric traits using summary data from large consortia, with sample sizes ranging from 151,013 to 806,834 individuals. Findings for genetically predicted ACLY inhibition were compared to those obtained for genetically predicted HMGCR inhibition using the same instrument selection strategy and outcome data. **Results:** Primary MR analyses showed that genetically predicted ACLY inhibition was associated with lower waist-to-hip ratio (β per 1 standard deviation lower LDL-c: −1.17; 95% confidence interval (CI): −1.61 to −0.73; *p* < 0.001) but not with risk of T2DM (odds ratio (OR) per standard deviation lower LDL-c: 0.74, 95% CI = 0.25 to 2.19, *p* = 0.59). In contrast, genetically predicted HMGCR inhibition was associated with higher waist-to-hip ratio (β = 0.15; 95%CI = 0.04 to 0.26; *p* = 0.008) and T2DM risk (OR = 1.73, 95% CI = 1.27 to 2.36, *p* < 0.001). The MR analyses considering secondary outcomes showed that genetically predicted ACLY inhibition was associated with a lower waist-to-hip ratio adjusted for body mass index (BMI) (β = −1.41; 95%CI = −1.81 to −1.02; *p* < 0.001). In contrast, genetically predicted HMGCR inhibition was associated with higher HbA1c (β = 0.19; 95%CI = 0.23 to 0.49; *p* < 0.001) and BMI (β = 0.36; 95%CI = 0.23 to 0.49; *p* < 0.001). **Conclusions:** Human genetic evidence supports the metabolically favourable effects of ACLY inhibition on body weight distribution, in contrast to HMGCR inhibition. These findings should be used to guide and prioritize ongoing clinical development efforts.

## 1. Introduction

Metabolic dysfunction is characterized by a cluster of shared risk factors that include obesity, insulin resistance, type 2 diabetes mellitus (T2DM), dyslipidaemia and atherosclerosis [1]. This is a growing concern as it significantly contributes to cardiovascular disease (CVD) morbidity and mortality worldwide [1]. Individuals with T2DM are at an increased risk of developing various vascular complications [2], and it is estimated that the number of people affected by T2DM will rise to 629 million by 2045 [3]. One of the primary drivers of CVD in individuals with metabolic dysfunction is atherogenic dyslipidaemia—a condition for which several lipid-lowering therapies have been developed, with statins being the most widely prescribed. While statins have been shown to be effective in the prevention of CVD events [4], their use is associated with adverse effects that include increased fasting glucose levels and glycosylated haemoglobin A1c (HbA1c), which can lead to a higher risk of developing T2DM [5,6]. In fact, both clinical trial and genetic data have supported that LDL-c lowering, particularly through HMGCR inhibition, may increase body weight and T2DM risk [7,8]. Despite existing efforts to combat atherosclerosis through lipid-lowering therapies, there is still a pressing need to develop more effective therapies that can reduce atherogenic CVD risk while minimizing potential adverse metabolic effects. 

Adenosine triphosphate-citrate lyase (ACLY) is an enzyme in the cholesterol biosynthesis pathway that functions upstream of 3-hydroxy-3-methylglutaryl-coenzyme A reductase (HMGCR), the enzyme targeted by statins. More specifically, ACLY catalyses the conversion of citrate into acetyl-coenzyme A—a key metabolite that functions in de novo fatty acids, cholesterol and coenzyme Q biosynthesis, all of which carry out essential cellular functions [9,10]. ACLY is expressed ubiquitously in human tissues and exhibits particularly greater expression levels in lipogenic tissues, including adipose and liver tissue [11]. Given the central role of ACLY in glucose and lipid metabolism, ACLY inhibition has emerged as a therapeutic strategy that is being pursued for cardiovascular and metabolic diseases. In fact, clinical trial data have shown that bempedoic acid, an ACLY inhibitor, reduces low-density lipoprotein cholesterol (LDL-c) levels and subsequently lowers cardiovascular disease (CVD) risk [12,13]. Interestingly, and in contrast to statins, preliminary evidence from observational and clinical trial data for bempedoic acid suggests that ACLY inhibition might have favourable metabolic effects [14,15]. The potential for ACLY inhibitors to provide metabolic benefits whilst lowering LDL-c levels when compared to its therapeutic counterparts is particularly important considering the rising incidence of metabolic diseases. Considering the effect of metabolic and glycaemic traits on CVD risk, it is essential to explore the potential discrepancies between different lipid-lowering drug targets with regard to their effects on metabolic disease risk [16]. 

Genetic variants predicting the perturbation of pharmacological targets can be used in the Mendelian randomization (MR) paradigm to rapidly and cost-effectively investigate on-target drug effects [17,18]. The random allocation of genetic variants at conception means that this approach is less vulnerable to bias from environmental confounding and reverse causation that can hinder causal inference in traditional epidemiological studies. Notably, previous MR analyses investigating the effects of ACLY inhibition used a genetic instrument that was not robustly associated with LDL-c and further considered T2DM risk as an outcome but not other related glycaemic or anthropometric traits [19,20,21]. Recent work has identified more robust genetic instruments for the effect of ACLY inhibition using variants related to *ACLY* gene expression and lower LDL-c levels [22]. Well-powered genetic data are also available for glycaemic traits and anthropometric traits, including body weight and body fat distribution [23,24]. 

In this study, we aimed to leverage genetic instruments that proxy ACLY inhibition in the MR paradigm to investigate the effects on metabolic and anthropometric outcomes. The insights gained from this study will inform the future clinical development of ACLY inhibitors and provide human causal evidence regarding the efficacy of this strategy for metabolic and anthropometric outcomes.

## 2. Materials and Methods

**Figure 1:**
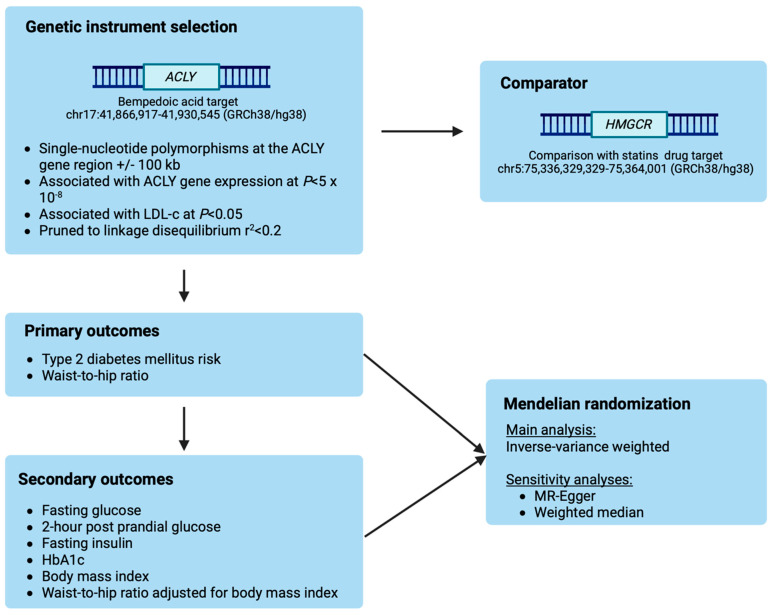
Study design overview. Created with BioRender.com on 1 July 2024.

Mendelian randomization is used to study the causal effect of an exposure on an outcome of interest by leveraging human genetic variation. This approach helps overcome the limitations of traditional epidemiological studies and can provide useful insights that inform randomized controlled trials. In this study, we used a two-sample MR approach to investigate potential anthropometric and metabolic effects of ACLY inhibition. We first identified genetic variants to proxy the effects of ACLY inhibition. Next, in primary analyses, we investigated the associations of genetically predicted ACLY inhibition with the primary outcomes of waist-to-hip ratio and T2DM risk. In secondary analyses, we extended our list of outcomes to include fasting glucose, 2 h post-prandial glucose, fasting insulin, glycated haemoglobin (HbA1c), waist-to-hip ratio adjusted for body mass index (BMI) and BMI. We conducted the same analyses for genetically predicted HMGCR, to serve as a comparator. An overview of the study design is schematically presented in Figure 1.

### 2.1. Selection of Genetic Instruments

As instruments for ACLY inhibition, we selected weakly correlated (pairwise r^2^ < 0.2 using the 1000G European Reference Panel) genetic variants within *±100 kB of* the *ACLY* gene region (*chr17:41,866,917-41,930,545*; GRCh38/hg38) associated with lower *ACLY* gene expression at *p* < 5 × 10^−8^ in blood samples from 31,684 individuals (eQTLGen, N = 31,684) [25] and also associated with lower circulating LDL-c at *p* < 0.01 in 1.65 million individuals using data from the Global Lipids Genetics Consortium (GLGC) [26]. As instruments for HMGCR inhibition, we considered variants from *±100 kB of* the *HMGCR* gene region (*chr5:75,336,329-75,364,001*; GRCh38/hg38) using the same strategy as for ACLY. 

### 2.2. Outcome Data Sources

Details prevailing to the genome-wide association study (GWAS) data sources used in primary and secondary MR analyses are presented in Table 1. Our primary outcomes included T2DM risk (N_cases_ = 180,834, N_controls_ = 1,159,055) using summary statistics from DIAMANTE [27] and waist-to-hip ratio using summary statistics from the Genetic Investigation of Anthropometric Traits (GIANT) Consortium (N = 694,649, standard deviation units). Outcomes selected for secondary analyses included fasting glucose (mmol/L), 2 h post-prandial glucose following an oral glucose tolerance test (mmol/L), fasting insulin (log-transformed pmol/L), glycated haemoglobin (HbA1c, %) from the Meta-Analyses of Glucose and Insulin-related traits Consortium (MAGIC, N = 200,622) [23], waist-to-hip ratio adjusted for BMI (GIANT, N = 694,649, standard deviation units) and BMI (GIANT, N = 806,834, standard deviation units).

### 2.3. Statistical Analyses

We conducted two-sample random-effects inverse-variance-weighted MR [29] as the main analysis to estimate the association of genetically predicted ACLY inhibition on our selected outcomes. MR is a statistical approach that uses genetic variants as instrumental variables to investigate the causal effects of an exposure on an outcome of interest. There are three main assumptions underlying the robust utilization of genetic instruments to facilitate MR. Firstly, the genetic instrument(s) must be associated with the exposure of interest (i.e., the relevance assumption). Secondly, no confounding factors influence the association between the genetic instrument(s) and outcomes of interest (i.e., the independence assumption). Thirdly, the genetic instrument is only related to the outcome via the exposure, ensuring the absence of pleiotropic effects that may bias MR estimates (i.e., the exclusion restriction assumption) [30]. For sensitivity analyses, we used the MR-Egger [31] and weighted median methods [32], as they make different assumptions about the presence of invalid instruments and pleiotropy [30]. As a sensitivity analysis to explore that any observed associations for genetically predicted ACLY inhibition are not attributable to correlation between the variants employed as instruments, we also tested the association of the lead variant in the ACLY inhibition instrument (based on association with gene expression) with the considered outcomes. 

### 2.4. Reporting

For continuous outcomes, we report estimated β and 95% confidence intervals (CI) for the putative effects of ACLY and HMGCR inhibition on primary and secondary outcomes of interest. β values represent the changes in the corresponding unit change described in Table 1 per standard deviation decrease in genetically predicted LDL-c levels. For binary outcomes, we report natural log odds ratios (ORs) and 95% CI per standard deviation decrease in genetically predicted LDL-c levels. A Bonferroni correction was made in ascertaining the statistical significance of the two primary outcomes. A separate Bonferroni correction was made in ascertaining statistical significance for the six secondary outcomes, which were considered exploratory in the context of at least one positive primary outcome.

## 3. Results

We conducted two-sample MR analyses to estimate associations between genetically predicted ACLY inhibition and anthropometric and metabolic traits. We compared the estimates with those for genetically proxied HMGCR inhibition, a clinically validated comparator. 

We leveraged five weakly correlated genetic instruments within the *ACLY* gene locus that were significantly associated with *ACLY* gene expression and LDL-c levels. Following the same rationale for the selection of genetic instruments, four genetic variants were identified as instruments for HMGCR inhibition. Genetic instruments and their associations with their respective gene expression and LDL-c levels are presented in Table 2 and Table 3.

**Figure 2:**
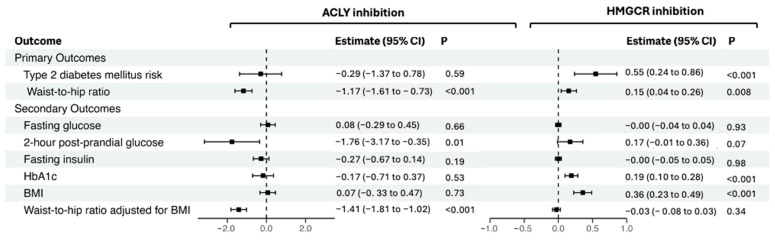
Mendelian randomization estimates per standard deviation lower LDL-c via ACLY and HMGCR inhibition on primary and secondary outcomes.

The main results from MR analyses are shown in Figure 2. In our primary MR analyses, we found that genetically predicted ACLY inhibition was associated with a lower waist-to-hip ratio (β = −1.17 per standard deviation lower LDL-c, 95% confidence intervals (CI)= −1.61 to −0.73), *p* < 0.001) but not with risk of T2DM (odds ratio (OR): 0.74, 95% confidence interval: 0.25 to 2.19, *p* = 0.593) (Table S1). In contrast, genetically predicted HMGCR inhibition was associated with a higher waist-to-hip ratio (β = 0.15; 95%CI = 0.04 to 0.26; *p* = 0.008) and T2DM risk (OR = 1.73, 95% CI = 1.27 to 2.36, *p* < 0.001). Consistent findings were generally obtained in statistical sensitivity analyses using the MR-Egger and weighted median methods, although with larger confidence intervals likely attributable to lower statistical power (Tables S1 and S2).

In secondary MR analyses, we found that genetically predicted ACLY inhibition was associated with a lower waist-to-hip ratio adjusted for BMI (β = −1.41; 95%CI = −1.81 to −1.02; *p* < 0.001). No strong association was found for genetically predicted ACLY and the other considered secondary outcomes (Table S1). In contrast, genetically predicted HMGCR inhibition was associated with higher HbA1c (β = 0.19; 95%CI = 0.23 to 0.49; *p* < 0.001) and BMI (β = 0.36; 95%CI = 0.23 to 0.49; *p* < 0.001) (Table S2).

Considering only the lead variant in the ACLY inhibition instrument (rs34200091 C allele), robust associations were observed with a lower waist-to-hip ratio (−0.011; 95%CI = −0.017 to −0.006; *p* < 0.001) and waist-to-hip ratio adjusted for BMI (−0.013; 95%CI = −0.018 to −0.008; *p* < 0.001) but not any of the other considered primary or secondary outcomes (Table S3).

## 4. Discussion

In this study, we identified genetic instruments to proxy ACLY inhibition and leveraged these in a two-sample MR design to investigate the effects of ACLY inhibition on metabolic and anthropometric outcomes. Our findings indicate that genetically predicted ACLY inhibition, which was used as a proxy for investigating pharmacological ACLY inhibition, is associated with a lower waist-to-hip ratio in our primary analyses and consistently with waist-to-hip ratio adjusted for BMI in our secondary analyses. No strong associations were found with any of the other considered metabolic traits. These findings contrast those for genetically predicted HMGCR inhibition, which corroborated clinical trial data to support the effects of statins on increasing T2DM risk, waist-to-hip ratio, HbA1c and BMI. These findings offer valuable insights for guiding and prioritizing ongoing clinical development efforts for lipid-lowering therapies.

Previous animal studies have shown that ACLY inhibition leads to improved metabolic health and physical strength in wild-type mice fed with a high-fat diet [14,33]. Bempedoic acid was also shown to reduce fasting glucose, fasting insulin and glucose intolerance in mouse models, suggesting improvements in insulin sensitivity [34]. There is suggestive evidence from clinical trials that these effects may translate to humans, as a meta-analysis of randomized trials suggests that bempedoic acid may reduce the incidence and progression of diabetes [35]. However, this was not replicated in the latest published Cholesterol Lowering via Bempedoic Acid, and ACL-Inhibiting Regimen (CLEAR) Outcomes trial, which showed that treatment with bempedoic acid for patients without diabetes had no effect on the risk of new-onset T2DM or HbA1c levels [15], in line with our findings. In contrast, preclinical studies have highlighted potential associations between ACLY inhibitors, including bempedoic acid and BMS-303141, and weight loss independently of alterations in food intake [12,34,36]. This is consistent with sub-analyses of clinical trials that have identified a link between ACLY inhibition and weight loss [37] and with our findings that support effects on body weight distribution.

MR can overcome the limitations of traditional epidemiological studies and animal studies, in that the paradigm uses human data to infer causal effects. However, previous MR studies on ACLY inhibition have had major limitations related to the selection of genetic instruments. For instance, Ference et al. selected genetic instruments on the basis of a wide genomic window of 500 kB and a very liberal *p*-value threshold of *p* < 0.05 that likely results in the incorporation of irrelevant instruments [19]. The genetic variants they selected additionally did not explain variability in any ACLY gene product and did not associate with LDL-c levels upon attempted replication and validation [20,21,38]. In this study, we selected genetic variants used as instrumental variables based on associations with lower *ACLY* gene expression at a genome-wide significance level and, additionally, with LDL-c at a nominal significance level. This step establishes the biological and clinical plausibility of our selected instruments and thereby strengthens the evidence from our subsequent MR findings. This approach has been adopted by other MR studies but with different outcomes under investigation. For instance, using this approach of selecting genetic instruments, Mohammadi-Shemirani showed that a genetically predicted reduction in ACLY expression was associated with reduced risk of chronic kidney disease but was not associated with estimated glomerular filtration rate and albumin-to-creatinine ratio [22]. 

There is significant interest in the potential adverse effects of different lipid-lowering therapies [39]. Our current MR findings shed further light on the differential effects of LDL-c lowering through HMGCR and ACLY inhibition, highlighted by the opposing directionality of their MR associations with some anthropometric traits and T2DM risk. ACLY inhibitors, that function at the intersection of fatty acid, cholesterol and carbohydrate metabolism, modulate lipid and glucose metabolism pathways, potentially leading to decreased waist-to-hip ratio. Conversely, our findings, together with prior clinical evidence, suggest that statins lead to increased T2DM liability via the modulation of glucose metabolism [8]. Recent data from randomized controlled trials supported that statin therapy increases blood glucose, which translates into an increased risk of T2DM and worsening glycaemic control among those with T2DM [39]. Despite this, the beneficial effects of statins on major vascular events are generally considered to outweigh the detrimental effects on glucose and energy metabolism. However, better lipid-lowering strategies, reducing major vascular events as well as improving glucose and energy metabolism have been lacking so far. Our current data together with those from clinical trials suggest that ACLY inhibition may be such a strategy [13]. 

Several mechanisms have been put forward to explain the role of statins in the impairment of insulin sensitivity, secretion, and subsequent development of T2DM. Previous genetic and trial data indicated that the elevated risk of diabetes associated with statin therapy may be, in part, attributable to an increase in body weight, which, in turn, increases the risk of developing diabetes [8]. Growing evidence supports that body fat distribution, rather than body weight per se, may be a mediating risk of developing T2DM [40,41]. The waist-to-hip ratio is used to clinically estimate visceral obesity [40,42], which in turn may induce insulin resistance [40,43] and also vascular disease [43,44]. We recently showed in the Diabetes Prevention Program and the Prediabetes Lifestyle Intervention Study that the weight-loss-induced remission of prediabetes to normal glucose tolerance was mediated by a reduction in visceral adipose tissue, with an associated reduction in the risk of developing T2DM [40,45,46]. In the context of data from our current study that supported favourable effects of ACLY inhibition on the waist-to-hip ratio, we hypothesize that by beneficially impacting body fat distribution, ACLY inhibition may contribute to reduced T2DM risk in clinical practice. Our current human genetic data also support that HMGCR inhibition, in contrast to ACLY inhibition, increases BMI, waist-to-hip ratio and T2DM risk. Similar results have been reported with statin treatment [39]. A potential explanation for this finding is that the inhibition of HMGCR in the liver results in the downregulation of the mevalonate pathway, increased LDL receptor (LDLR) expression and a reduction in LDL-c concentrations. This may lead to impaired insulin secretion in the pancreas and increased T2DM risk [47,48]. 

This work has several strengths. Firstly, and as previously discussed, we employed a robust approach to select genetic instruments that are biologically plausible proxies for ACLY inhibition, supporting the validity of our MR findings. Secondly, we harnessed data from large-scale GWAS datasets and extended our outcomes to include additional markers of metabolic function than previously studied. Thirdly, we contrasted with a clinically validated comparator, the statin drug target HMGCR.

Our results should also be interpreted in the context of their limitations. Firstly, the majority of publicly available GWAS used in this study pertains to populations of European ancestry, thereby limiting the generalizability of our results to populations of other ancestries. Caution should, therefore, be taken when interpreting these findings in the context of populations of diverse genetic ancestry. This also highlights the need to generate genetic association studies in diverse population groups to allow for investigation into whether these findings extrapolate to the broader global population. Secondly, MR analyses consider the associations of genetic variants predicting small lifelong changes of drug target inhibition, which differ from discrete clinical interventions of larger magnitude in later life. Therefore, MR estimates should not be extrapolated to infer the magnitude of effect association with a clinical intervention but, rather, should be used to shed light on the presence and direction of any effects. Moreover, some of the null observed associations may be false-negative findings due to inadequate statistical power. MR analyses can also be subject to bias from pleiotropic associations of the genetic variants, a possibility that cannot be entirely excluded despite our incorporation of statistical sensitivity analyses that are more robust to this. Finally, our study’s reliance on publicly available GWAS data may introduce biases. These limitations highlight the need for further investigations using large independent genetic association datasets, along with clinical studies, to validate our findings.

## 5. Conclusions

In conclusion, this human genetic evidence supports the metabolically favourable effects of ACLY inhibition on body weight distribution, in contrast to those observed for HMGCR inhibition. These findings should be used to inform and prioritize ongoing clinical development efforts and facilitate a discussion about which therapies to favour for the treatment of hypercholesterolemia in certain patient groups, such as those at high risk of developing T2DM.

## Figures and Tables

**Table 1:** Data sources and genome-wide association studies used for exposures, primary and secondary outcomes.

	Trait and Ascertainment	N	Units	Reference
**Exposure**	*ACLY* gene expression in blood	31,684	Standard deviation	Nature Genetics. 2021 Sep; 53(9), 1300–1310 [25]
	Low-density lipoprotein cholesterol	1,231,289	Standard deviation	Nature. 2021 Dec; 600(7890):675–679 [26]
**Primary outcomes**	Type 2 diabetes mellitus risk	N_cases_ = 180,834 N_controls_ = 1,159,055	Natural log-transformed OR	Nat Genet. 2022 May; 54(5):560–572 [27]
	Waist-to-hip ratio	697,734	Standard deviation	Hum Mol Genet. 2019 Jan; 28(1):166–174 [24]
**Secondary outcomes**	Fasting glucose—individuals without diabetes	200,622	mmol/L	Nat Genet. 2021 Jun; 53(6):840–860 [23]
	2-h post-prandial glucose—individuals without diabetes	63,396	mmol/L	Nat Genet. 2021 Jun; 53(6):840–860 [23]
	Fasting insulin—individuals without diabetes	151,013	Natural log-transformed pmol/L	Nat Genet. 2021 Jun; 53(6):840–860 [23]
	HbA1c	344,182	Standard deviation	UK Biobank—Neale Lab [28]
	Waist-to-hip ratio adjusted for body mass index	694,649	Standard deviation	Hum Mol Genet. 2019 Jan 1; 28(1):166–174 [24]
	Body mass index	806,834	Standard deviation	

**Table 2:** Genetic variants selected as instruments for ACLY inhibition and their associations with gene expression and low-density lipoprotein cholesterol.

					*ACLY* Gene Expression Associations		LDL-c Associations		
Variant	Chromosome	Position (hg19)	Effect Allele	Other Allele	Z-Score	*p* Value	β	Standard Error	*p* Value
rs6503666	17	39954291	G	A	−7.663	1.80 × 10^−14^	−0.005	0.002	0.003
rs4796707	17	39964930	C	T	−11.142	7.80 × 10^−29^	−0.008	0.001	9.93 × 10^−08^
rs34200091	17	40014216	A	G	−17.964	3.70 × 10^−72^	−0.010	0.002	9.91 × 10^−07^
rs76162894	17	40085165	A	C	−6.452	1.10 × 10^−10^	−0.022	0.008	0.005
rs2070106	17	40125864	G	A	−6.442	1.18 × 10^−10^	−0.004	0.002	0.009

**Table 3:** Genetic variants selected as instruments for HMGCR inhibition and their associations with gene expression and low-density lipoprotein cholesterol.

					*HMGCR* Gene Expression Associations		LDL-c Associations		
Variant	Chromosome	Position (hg19)	Effect Allele	Other Allele	Z-Score	*p* Value	β	Standard Error	*p* Value
rs13356603	5	74569357	C	T	−6.909	4.90 × 10^−12^	−0.068	0.002	<1.00 × 10^−300^
rs114760090	5	74622241	G	A	−7.840	4.50 × 10^−15^	−0.017	0.003	7.53 × 10^−09^
rs6453133	5	74692776	A	G	−14.967	1.21 × 10^−50^	−0.050	0.002	4.24 × 10^−229^
rs151000110	5	74725216	G	A	−8.659	4.77 × 10^−18^	−0.067	0.003	1.65 × 10^−103^

## Data Availability

The summary data used in these analyses can be retrieved from the primary studies, which are cited. All statistical code used in this work can be obtained upon reasonable request to the corresponding author.

## Supplementary Materials

The following supporting information can be downloaded at: https://www.mdpi.com/article/10.3390/genes15081059/s1, Table S1: Mendelian randomization statistical sensitivity analyses for ACLY inhibition. Beta values represent the change per standard deviation increase in genetically predicted LDL-c levels (with units as per the main Table 1), and for binary outcomes, we report natural log odds ratios. Table S2: Mendelian randomization statistical sensitivity analyses for HMGCR inhibition. Beta values represent the change per standard deviation increase in genetically predicted LDL-c levels (with units as per the main Table 1), and for binary outcomes, we report natural log odds ratios (ORs). Table S3: Associations of the lead variant in the ACLY inhibition instrument (rs34200091) with the considered primary and secondary outcomes. Beta values represent the association per LDL-c decreasing allele (with units as per the main Table 1), and for binary outcomes, we report natural log odds ratios.

## Abbreviations

ACLY	adenosine triphosphate-citrate lyase
BMI	body mass index
CVD	cardiovascular disease
GWAS	genome-wide association study
HbA1c	glycated haemoglobin
HMGCR	3-hydroxy-3-methylglutaryl-coenzyme A reductase
LDL-c	low-density lipoprotein cholesterol
MR	Mendelian randomization
OR	odds ratio
T2DM	type 2 diabetes mellitus

## References

[1] Alberti K.G., Zimmet P., Shaw J. (2006). Metabolic syndrome—A new world-wide definition. A Consensus Statement from the International Diabetes Federation. Diabet. Med..

[2] American Diabetes Association Professional Practice Committee (2022). 16. Diabetes Care in the Hospital: Standards of Medical Care in Diabetes-2022. Diabetes Care.

[3] Regufe V.M.G., Pinto C., Perez P. (2020). Metabolic syndrome in type 2 diabetic patients: A review of current evidence. Porto Biomed. J..

[4] Collins R., Reith C., Emberson J., Armitage J., Baigent C., Blackwell L., Blumenthal R., Danesh J., Smith G.D., DeMets D. (2016). Interpretation of the evidence for the efficacy and safety of statin therapy. Lancet.

[5] Chogtu B., Magazine R., Bairy K.L. (2015). Statin use and risk of diabetes mellitus. World J. Diabetes.

[6] Agarwala A., Kulkarni S., Maddox T. (2018). The Association of Statin Therapy with Incident Diabetes: Evidence, Mechanisms, and Recommendations. Curr. Cardiol. Rep..

[7] Wu P., Moon J.Y., Daghlas I., Franco G., Porneala B.C., Ahmadizar F., Richardson T.G., Isaksen J.L., Hindy G., Yao J. (2022). Obesity Partially Mediates the Diabetogenic Effect of Lowering LDL Cholesterol. Diabetes Care.

[8] Swerdlow D.I., Preiss D., Kuchenbaecker K.B., Holmes M.V., Engmann J.E., Shah T., Sofat R., Stender S., Johnson P.C., Scott R.A. (2015). HMG-coenzyme A reductase inhibition, type 2 diabetes, and bodyweight: Evidence from genetic analysis and randomised trials. Lancet.

[9] Pietrocola F., Galluzzi L., Bravo-San Pedro J.M., Madeo F., Kroemer G. (2015). Acetyl coenzyme A: A central metabolite and second messenger. Cell Metab..

[10] Willmes D.M., Kurzbach A., Henke C., Schumann T., Zahn G., Heifetz A., Jordan J., Helfand S.L., Birkenfeld A.L. (2018). The longevity gene INDY (I’m Not Dead Yet) in metabolic control: Potential as pharmacological target. Pharmacol. Ther..

[11] Pinkosky S.L., Groot P.H.E., Lalwani N.D., Steinberg G.R. (2017). Targeting ATP-Citrate Lyase in Hyperlipidemia and Metabolic Disorders. Trends Mol. Med..

[12] Pinkosky S.L., Newton R.S., Day E.A., Ford R.J., Lhotak S., Austin R.C., Birch C.M., Smith B.K., Filippov S., Groot P.H.E. (2016). Liver-specific ATP-citrate lyase inhibition by bempedoic acid decreases LDL-C and attenuates atherosclerosis. Nat. Commun..

[13] Nissen S.E., Lincoff A.M., Brennan D., Ray K.K., Mason D., Kastelein J.J.P., Thompson P.D., Libby P., Cho L., Plutzky J. (2023). Bempedoic Acid and Cardiovascular Outcomes in Statin-Intolerant Patients. N. Engl. J. Med..

[14] Morrow M.R., Batchuluun B., Wu J., Ahmadi E., Leroux J.M., Mohammadi-Shemirani P., Desjardins E.M., Wang Z., Tsakiridis E.E., Lavoie D.C.T. (2022). Inhibition of ATP-citrate lyase improves NASH, liver fibrosis, and dyslipidemia. Cell Metab..

[15] Ray K.K., Nicholls S.J., Li N., Louie M.J., Brennan D., Lincoff A.M., Nissen S.E., Committees C.O. (2024). Efficacy and safety of bempedoic acid among patients with and without diabetes: Prespecified analysis of the CLEAR Outcomes randomised trial. Lancet Diabetes Endocrinol..

[16] Kosmas C.E., Silverio D., Sourlas A., Garcia F., Montan P.D., Guzman E. (2018). Impact of lipid-lowering therapy on glycemic control and the risk for new-onset diabetes mellitus. Drugs Context.

[17] Burgess S., Mason A.M., Grant A.J., Slob E.A.W., Gkatzionis A., Zuber V., Patel A., Tian H., Liu C., Haynes W.G. (2023). Using genetic association data to guide drug discovery and development: Review of methods and applications. Am. J. Hum. Genet..

[18] Gill D., Georgakis M.K., Walker V.M., Schmidt A.F., Gkatzionis A., Freitag D.F., Finan C., Hingorani A.D., Howson J.M.M., Burgess S. (2021). Mendelian randomization for studying the effects of perturbing drug targets. Wellcome Open Res..

[19] Ference B.A., Ray K.K., Catapano A.L., Ference T.B., Burgess S., Neff D.R., Oliver-Williams C., Wood A.M., Butterworth A.S., Di Angelantonio E. (2019). Mendelian Randomization Study of ACLY and Cardiovascular Disease. N. Engl. J. Med..

[20] Klarin D., O’Donnell C.J., Kathiresan S. (2020). Mendelian Randomization Study of ACLY and Cardiovascular Disease. N. Engl. J. Med..

[21] Holm H., Sulem P., Helgadottir A., Tragante V., Thornorleifsson G., Guethbjartsson D., Stefansson K. (2020). Mendelian Randomization Study of ACLY and Cardiovascular Disease. N. Engl. J. Med..

[22] Mohammadi-Shemirani P., Chong M., Perrot N., Pigeyre M., Steinberg G.R., Pare G., Krepinsky J.C., Lanktree M.B. (2022). ACLY and CKD: A Mendelian Randomization Analysis. Kidney Int. Rep..

[23] Chen J., Spracklen C.N., Marenne G., Varshney A., Corbin L.J., Luan J., Willems S.M., Wu Y., Zhang X., Horikoshi M. (2021). The trans-ancestral genomic architecture of glycemic traits. Nat. Genet..

[24] Pulit S.L., Stoneman C., Morris A.P., Wood A.R., Glastonbury C.A., Tyrrell J., Yengo L., Ferreira T., Marouli E., Ji Y. (2019). Meta-analysis of genome-wide association studies for body fat distribution in 694 649 individuals of European ancestry. Hum. Mol. Genet..

[25] Vosa U., Claringbould A., Westra H.J., Bonder M.J., Deelen P., Zeng B., Kirsten H., Saha A., Kreuzhuber R., Yazar S. (2021). Large-scale cis- and trans-eQTL analyses identify thousands of genetic loci and polygenic scores that regulate blood gene expression. Nat. Genet..

[26] Graham S.E., Clarke S.L., Wu K.H., Kanoni S., Zajac G.J.M., Ramdas S., Surakka I., Ntalla I., Vedantam S., Winkler T.W. (2021). The power of genetic diversity in genome-wide association studies of lipids. Nature.

[27] Mahajan A., Spracklen C.N., Zhang W., Ng M.C.Y., Petty L.E., Kitajima H., Yu G.Z., Rueger S., Speidel L., Kim Y.J. (2022). Multi-ancestry genetic study of type 2 diabetes highlights the power of diverse populations for discovery and translation. Nat. Genet..

[28] Neale B. 2018. http://www.nealelab.is/uk-biobank/.

[29] Burgess S., Butterworth A., Thompson S.G. (2013). Mendelian randomization analysis with multiple genetic variants using summarized data. Genet. Epidemiol..

[30] Davies N.M., Holmes M.V., Davey Smith G. (2018). Reading Mendelian randomisation studies: A guide, glossary, and checklist for clinicians. BMJ.

[31] Bowden J., Davey Smith G., Burgess S. (2015). Mendelian randomization with invalid instruments: Effect estimation and bias detection through Egger regression. Int. J. Epidemiol..

[32] Bowden J., Davey Smith G., Haycock P.C., Burgess S. (2016). Consistent Estimation in Mendelian Randomization with Some Invalid Instruments Using a Weighted Median Estimator. Genet. Epidemiol..

[33] Sola-Garcia A., Caliz-Molina M.A., Espadas I., Petr M., Panadero-Moron C., Gonzalez-Moran D., Martin-Vazquez M.E., Narbona-Perez A.J., Lopez-Noriega L., Martinez-Corrales G. (2023). Metabolic reprogramming by Acly inhibition using SB-204990 alters glucoregulation and modulates molecular mechanisms associated with aging. Commun. Biol..

[34] Pinkosky S.L., Filippov S., Srivastava R.A., Hanselman J.C., Bradshaw C.D., Hurley T.R., Cramer C.T., Spahr M.A., Brant A.F., Houghton J.L. (2013). AMP-activated protein kinase and ATP-citrate lyase are two distinct molecular targets for ETC-1002, a novel small molecule regulator of lipid and carbohydrate metabolism. J. Lipid Res..

[35] Masson W., Lobo M., Lavalle-Cobo A., Masson G., Molinero G. (2020). Effect of bempedoic acid on new onset or worsening diabetes: A meta-analysis. Diabetes Res. Clin. Pract..

[36] Cramer C.T., Goetz B., Hopson K.L., Fici G.J., Ackermann R.M., Brown S.C., Bisgaier C.L., Rajeswaran W.G., Oniciu D.C., Pape M.E. (2004). Effects of a novel dual lipid synthesis inhibitor and its potential utility in treating dyslipidemia and metabolic syndrome. J. Lipid Res..

[37] Banach M., Duell P.B., Gotto A.M., Laufs U., Leiter L.A., Mancini G.B.J., Ray K.K., Flaim J., Ye Z., Catapano A.L. (2020). Association of Bempedoic Acid Administration with Atherogenic Lipid Levels in Phase 3 Randomized Clinical Trials of Patients with Hypercholesterolemia. JAMA Cardiol..

[38] Damask A., Paulding C., Baras A., Carey D., Abecasis G.R. (2020). Mendelian Randomization Study of ACLY and Cardiovascular Disease. N. Engl. J. Med..

[39] Reith C., Preiss D., Blackwell L., Emberson J., Spata E., Davies K., Halls H., Holland L., Wilson K., Cholesterol Treatment Trialists’ Collaboration (2024). Effects of statin therapy on diagnoses of new-onset diabetes and worsening glycaemia in large-scale randomised blinded statin trials: An individual participant data meta-analysis. Lancet Diabetes Endocrinol..

[40] Sandforth A., von Schwartzenberg R.J., Arreola E.V., Hanson R.L., Sancar G., Katzenstein S., Lange K., Preissl H., Dreher S.I., Weigert C. (2023). Mechanisms of weight loss-induced remission in people with prediabetes: A post-hoc analysis of the randomised, controlled, multicentre Prediabetes Lifestyle Intervention Study (PLIS). Lancet Diabetes Endocrinol..

[41] Stefan N., Schulze M.B. (2023). Metabolic health and cardiometabolic risk clusters: Implications for prediction, prevention, and treatment. Lancet Diabetes Endocrinol..

[42] Schulze M.B., Thorand B., Fritsche A., Haring H.U., Schick F., Zierer A., Rathmann W., Kroger J., Peters A., Boeing H. (2012). Body adiposity index, body fat content and incidence of type 2 diabetes. Diabetologia.

[43] Bluher M. (2019). Obesity: Global epidemiology and pathogenesis. Nat. Rev. Endocrinol..

[44] Despres J.P. (2012). Body fat distribution and risk of cardiovascular disease: An update. Circulation.

[45] Jumpertz von Schwartzenberg R., Vazquez Arreola E., Sandforth A., Hanson R.L., Birkenfeld A.L. (2024). Role of weight loss-induced prediabetes remission in the prevention of type 2 diabetes: Time to improve diabetes prevention. Diabetologia.

[46] Birkenfeld A.L., Mohan V. (2024). Prediabetes remission for type 2 diabetes mellitus prevention. Nat. Rev. Endocrinol..

[47] Laakso M., Fernandes Silva L. (2023). Statins and risk of type 2 diabetes: Mechanism and clinical implications. Front. Endocrinol..

[48] Dannecker C., Wagner R., Peter A., Hummel J., Vosseler A., Haring H.U., Fritsche A., Birkenfeld A.L., Stefan N., Heni M. (2021). Low-Density Lipoprotein Cholesterol Is Associated with Insulin Secretion. J. Clin. Endocrinol. Metab..

