# Supplementary material for: Effects of ACLY Inhibition on Body Weight Distribution: A Drug Target Mendelian Randomization Study
Source: Genes (Basel). 2024 Aug 12;15(8):1059. doi: 10.3390/genes15081059 (PMC11353272; doi:10.3390/genes15081059)
Supplement: Supplementary file 1 [file genes-15-01059-s001.zip › genes-3126996-supplementary.pdf]

Effects of ACLY inhibition on body weight distribution: a drug target Mendelian randomization study

Supplementary Material

Dipender Gill MD PhD[1,2]*, Marie-Joe Dib PhD[3], Rubinder Gill MSc[2], Stefan R. Bornstein[4-6], Stephen Burgess PhD[7,8], Andreas L. Birkenfeld MD[5,9,10]

1. Department of Epidemiology and Biostatistics, School of Public Health, Imperial College London, London, UK.
2. Primula Group Ltd, London, UK.
3. Division of Cardiovascular Medicine, Perelman School of Advanced Medicine, University of Pennsylvania, PA, USA.
4. Department of Internal Medicine III, University Clinic, Technical University Dresden, Dresden, Germany
5. German Center for Diabetes Research (DZD), Neuherberg, Germany
6. Department of Diabetes, School of Cardiovascular and Metabolic Medicine & Sciences, King's College London, London, UK
7. MRC Integrative Epidemiology Unit, University of Bristol, Bristol, UK.
8. Department of Public Health and Primary Care, University of Cambridge, Cambridge, UK.
9. Department of Internal Medicine IV, Diabetology, Endocrinology and Nephrology, Eberhard Karls University Tübingen, Tübingen, Germany.
10. Institute for Diabetes Research and Metabolic Diseases, Helmholtz Center Munich, Eberhard Karls University Tübingen, Tübingen, Germany.

*Correspondence to Dr Dipender Gill, Department of Epidemiology and Biostatistics, School of Public Health, Imperial College London, London, W2 1PG, United Kingdom. Telephone: +44 7904843810. E-mail: dipender.gill@imperial.ac.uk

Table S1: Mendelian randomization statistical sensitivity analyses for ACLY inhibition. Beta values represent the change per standard deviation increase in genetically predicted LDL-c levels (with units as per the main Table 1), and for binary outcomes we report natural log odds ratios.

Outcome	Method	Beta	Standard error	P value
Type 2 diabetes mellitus risk	Inverse-variance weighted	0.294	0.549	0.593
Type 2 diabetes mellitus risk	Weighted median	0.821	0.662	0.215
Type 2 diabetes mellitus risk	Egger	1.465	1.569	0.35
Type 2 diabetes mellitus risk	Egger intercept	-0.009	0.011	0.422
Waist-to-hip ratio	Inverse-variance weighted	1.170	0.223	<0.001
Waist-to-hip ratio	Weighted median	1.207	0.266	<0.001
Waist-to-hip ratio	Egger	0.562	0.579	0.332
Waist-to-hip ratio	Egger intercept	0.005	0.004	0.257
Fasting glucose	Inverse-variance weighted	-0.081	0.189	0.668
Fasting glucose	Weighted median	-0.143	0.191	0.453
Fasting glucose	Egger	-0.724	0.469	0.122
Fasting glucose	Egger intercept	0.005	0.003	0.142
2-hour post-prandial glucose	Inverse-variance weighted	1.760	0.720	0.014
2-hour post-prandial glucose	Weighted median	1.664	0.891	0.062
2-hour post-prandial glucose	Egger	0.720	2.041	0.724
2-hour post-prandial glucose	Egger intercept	0.008	0.015	0.586
Fasting insulin	Inverse-variance weighted	0.265	0.205	0.197
Fasting insulin	Weighted median	0.117	0.220	0.595
Fasting insulin	Egger	0.492	0.656	0.453
Fasting insulin	Egger intercept	-0.002	0.005	0.711
HbA1c	Inverse-variance weighted	0.172	0.274	0.531
HbA1c	Weighted median	-0.201	0.249	0.418
HbA1c	Egger	-0.253	0.813	0.756

HbA1c	Egger intercept	0.003	0.006	0.574
Body mass index	Inverse-variance weighted	-0.068	0.203	0.737
Body mass index	Weighted median	0.082	0.192	0.671
Body mass index	Egger	-0.458	0.573	0.424
Body mass index	Egger intercept	0.003	0.004	0.463
Waist-to-hip ratio adjusted for body mass index	Inverse-variance weighted	1.414	0.201	<0.001
Waist-to-hip ratio adjusted for body mass index	Weighted median	1.388	0.284	<0.001
Waist-to-hip ratio adjusted for body mass index	Egger	0.832	0.504	0.099
Waist-to-hip ratio adjusted for body mass index	Egger intercept	0.004	0.004	0.214

Table S2: Mendelian randomization statistical sensitivity analyses for HMGCR inhibition. Beta values represent the change per standard deviation increase in genetically predicted LDL-c levels (with units as per the main Table 1), and for binary outcomes we report natural log odds ratios (ORs).

Outcome	Method	Beta	Standard error	P value
Type 2 diabetes mellitus risk	Inverse-variance weighted	-0.546	0.158	<0.001
Type 2 diabetes mellitus risk	Weighted median	-0.492	0.112	<0.001
Type 2 diabetes mellitus risk	Egger	0.322	0.323	0.319
Type 2 diabetes mellitus risk	Egger intercept	-0.051	0.018	0.005
Waist-to-hip ratio	Inverse-variance weighted	-0.152	0.057	0.008
Waist-to-hip ratio	Weighted median	-0.140	0.036	<0.001
Waist-to-hip ratio	Egger	0.154	0.154	0.315
Waist-to-hip ratio	Egger intercept	-0.018	0.009	0.039
Fasting glucose	Inverse-variance weighted	0.002	0.020	0.934
Fasting glucose	Weighted median	0.002	0.023	0.939
Fasting glucose	Egger	-0.091	0.074	0.218
Fasting glucose	Egger intercept	0.006	0.004	0.192
2-hour post-prandial glucose	Inverse-variance weighted	-0.172	0.095	0.07
2-hour post-prandial glucose	Weighted median	-0.160	0.104	0.125
2-hour post-prandial glucose	Egger	-0.275	0.334	0.41
2-hour post-prandial glucose	Egger intercept	0.006	0.019	0.747
Fasting insulin	Inverse-variance weighted	0.000	0.023	0.989
Fasting insulin	Weighted median	0.001	0.026	0.964
Fasting insulin	Egger	-0.001	0.084	0.994
Fasting insulin	Egger intercept	0.000	0.005	0.99
HbA1c	Inverse-variance weighted	-0.190	0.048	<0.001
HbA1c	Weighted median	-0.192	0.035	<0.001
HbA1c	Egger	0.044	0.128	0.73
HbA1c	Egger intercept	-0.014	0.007	0.057

Body mass index	Inverse-variance weighted	-0.357	0.067	<0.001
Body mass index	Weighted median	-0.292	0.046	<0.001
Body mass index	Egger	0.026	0.160	0.872
Body mass index	Egger intercept	-0.022	0.009	0.013
Waist-to-hip ratio adjusted for body mass index	Inverse-variance weighted	0.028	0.029	0.343
Waist-to-hip ratio adjusted for body mass index	Weighted median	0.027	0.029	0.336
Waist-to-hip ratio adjusted for body mass index	Egger	0.164	0.095	0.084
Waist-to-hip ratio adjusted for body mass index	Egger intercept	-0.008	0.005	0.136

Table S3: Associations of the lead variant in the ACLY inhibition instrument (rs34200091) with the considered primary and secondary outcomes. Beta values represent the association per LDL-c decreasing allele (with units as per the main Table 1), and for binary outcomes we report natural log odds ratios.

Outcome	Beta	Standard error	P value
Type 2 diabetes risk	-0.0077	0.0086	0.3698
Waist-to-hip ratio	-0.0114	0.0027	2.041e-05
Fasting glucose	0.0036	0.0024	0.0323
2-hour post-prandial glucose	-0.0171	0.0113	0.2356
Fasting insulin	-0.0017	0.0027	0.8863
HbA1c	0.0020	0.0030	0.5232
Body mass index	-0.0009	0.0026	0.7423
Waist-to-hip ratio adjusted for body mass index	-0.0131	0.0027	1.015e-06
